# “Too shocked to search” The COVID-19 shutdowns’ impact on the search for apprenticeships

**DOI:** 10.1186/s41937-021-00075-z

**Published:** 2021-09-30

**Authors:** Daniel Goller, Stefan C. Wolter

**Affiliations:** 1 Centre for Research in Economics of Education, University of Bern, Bern, Switzerland; 2 Swiss Coordination Centre for Research in Education, Aarau, Switzerland; 3 CESifo, Munich, Germany; 4 IZA, Bonn, Germany; 5 Swiss Institute for Empirical Economic Research, University of St. Gallen, St. Gallen, Switzerland

**Keywords:** COVID-19, Switzerland, Stringency Index, Apprenticeship, Vacancies, I20, J22

## Abstract

Even though the recession in Switzerland triggered by COVID-19 ultimately remained without consequences for the apprenticeship market, significantly fewer apprenticeship contracts had been signed in the months of the first shutdown in 2020 than in the same months of the previous year. Using daily search queries on the national administrative platform for apprenticeship vacancies from February 2020 until April 2021 as a proxy for the supply of potential apprentices, we find a temporal pattern that coincides perfectly with the development of signed apprenticeship contracts. Furthermore, the analyses show that the initially very strong relationship between the intensity of the politically imposed restrictions to fight the COVID-19 pandemic and the daily search queries diminished over time, leading to a search intensity in March 2021 that was back at pre-pandemic level.

## Motivation

Switzerland is proud of its well-functioning vocational education and training system (VET). Training young people specifically for the demands of a profession, as well as the needs of a company, has proven to be a good remedy against youth unemployment and counteracts the shortage of skilled workers (e.g., Bolli et al., 2021). Furthermore, apprentices benefit from the system as well, as they earn theoretical knowledge and working experience while they are trained to become professionals in the respective job.

However, all these advantages are also counterbalanced by the potential disadvantage that the demand for apprentices depends on the economic situation and does not simply follow the demographic trend of young people looking for post-compulsory education, as is the case with school-based types of education (for an overview on the effects of business cycles on apprenticeships see e.g., Brunello, 2009 or Muehlemann & Wolter, 2021 and for a general overview on the economics of apprenticeship see e.g., Wolter & Ryan, 2011 or Muehlemann & Wolter, 2020). Basically, the apprenticeship market consists of two sides, firms on the one side, demanding apprentices and on the other side the supply of young people willing to learn an occupation. The total number of training contracts therefore reflects the equilibrium of the number of training positions offered by firms willing to train, as well as the number of individuals willing to learn a job.

This work investigates the supply of apprentices during the COVID-19 pandemic as the potential reason for a temporal decrease in signed training contracts in the first months of the pandemic. Using an innovative data source, we are able to track the supply side in form of revealed preferences on a daily basis by the number of search queries on the national Swiss platform for apprenticeship vacancies. Although this does not show the supply of apprenticeship seekers as such, it does show the search intensity, which could not automatically be derived from the number of registered apprenticeship seekers.

In the past it was only possible to make assumptions as to whether it was supply or demand that primarily determined the number of apprenticeship contracts concluded, due to the very limited availability of data on supply and demand or even the complete absence of such data, as is the situation in Switzerland. In the case of large demographic fluctuations, it was assumed, *ceteris paribus*, that changes in contracts were due to the influence of supply and, conversely, in the case of economic fluctuations, that the influence was due to changes in the demand for apprentices. Thus, this study contributes to a better understanding of the functioning of the apprenticeship market in Switzerland, as for the first time the supply of apprenticeship seekers can be tracked over time.

After the outbreak of the COVID-19 pandemic, there were fears, based on experiences in previous crises (Luethi & Wolter, 2020), that the apprenticeship market in Switzerland would primarily suffer as a result of the expected economic recession and its impact on the demand for apprentices. In April and May, the number of signed apprenticeship contracts fell significantly compared to the previous year's values. However, this number returned to the previous year's values later in the summer, so that when the apprenticeships of the 2020 cohort began, the same number of apprentices started an apprenticeship as before the pandemic. As there was no indication that the demand for apprentices changed significantly in the aftermath of the March 2020 shutdown and it is unlikely that apprenticeship vacancies fluctuate largely from month to month,^1^ we direct the focus in this paper to the supply of potential apprentices as an explanation for the observed temporal pattern of signed contracts.

Analysing over 10 million search queries for apprenticeship positions from end of February 2020 until April 2021, we find a sharp decrease of up to 40% in search queries during the first shutdown followed by a catch-up effect afterwards. Further, we find that the effect of politically imposed restrictions on the search intensity decreases over time. Consequently, we find a lower impact of the second shutdown starting in December 2020 until February 2021 on the intensity of searching for an apprenticeship. The empirical results show that the temporal pattern of the progression of the cumulative number of signed apprenticeship contracts is very similar to the pattern of the search intensity for apprenticeship positions. Even though it is not possible to postulate causal relationships based on the available data, the analyses show that the development of the apprenticeship market in Switzerland in the short run and in the wake of the COVID-19 pandemic was probably determined by a reaction of supply.

The rest of this work is structured as follows. We provide a short review of emerging literature on COVID-19 effects in general and the effects on the labour market and job search behaviour, in Sect. 2. Section 3 provides a short overview of the apprenticeship market in Switzerland in 2020. Section 4 introduces the methodology, data base and some descriptive statistics. Section 5 presents the results of the analysis, followed by concluding remarks in Sect. 6.

## Literature

There is a fast-growing COVID-19-related literature investigating economic consequences of the pandemic (compare e.g., Baker et al., 2020; Chetty et al., 2020; Goolsbee & Syverson, 2021; among many others). For Switzerland, an array of papers was published documenting the COVID-19 impact,^2^ often using very novel data available on an hourly, daily, weekly, or monthly base. The outcomes included new measures of GDP (Burri & Kaufmann, 2020), mobility and sales (Eckert & Mikosch, 2020; Persson et al., 2021), trade (Buechel et al., 2020), or shifts in retail payments (Kraenzlin et al., 2020).

There is also a growing literature on the effects of the pandemic on the labour market (e.g., Gupta et al., 2020; Forsythe et al., 2020; Coibion et al., 2020; among others) and more specifically on job search, which is a topic that comes closest to this paper. For the Swiss labour market Lalive et al. (2020) found that jobseekers during the crisis invested less time in their job search than before the Corona crisis. This was driven by a decline in the number of vacancies, as well as due to being afraid of becoming infected with the virus during the recruitment process. In another study using Dutch survey data, Balgova et al. (2021) found that unemployed individuals reduced their effort in searching for a job, while employed individuals searched rather more intensively. Finally, Marinescu et al. (2021) found job applications during the COVID-19 pandemic to decrease. They further found this to be related to increased unemployment benefits. This relationship weakened as the number of job applications increased again starting in May 2020.

The above-described implications and observations for the labour market in general can also be found in the apprenticeship market, even though the conditions for potential apprentices and the objectives of companies are different than in the case of regular workers. In an early approach to predict the supply and demand side of the German apprenticeship market in the first year of the COVID-19 crisis, Muehlemann et al. (2020) estimated, in contrast to the Swiss market, a COVID-19 induced 6% reduction in signed apprenticeship positions in 2020 in Germany. Similar predictions with different data sets and methods were made by Maier (2020) predicting a reduction in signed apprenticeship contracts in Germany induced by a simultaneous decrease in supply and demand and Oeynhausen et al. (2020) confirming, that indeed during the COVID-19 crisis there was a substantial decrease in firms’ demand for apprentices but also applications sent to companies and consequently a reduction also in the number of signed contracts in the official German statistics. The German studies have the possibility to rely on proxies for the potential supply of new apprentices by using data on the registered applicants in the data base of the Federal Employment Agency. A data set that does not exist in Switzerland as apprenticeship seekers cannot register in an official register.

## The apprenticeship market in Switzerland in 2020

There are no official data sources in Switzerland that would have allowed the supply of and demand for apprenticeships to be monitored periodically and at high frequency. However, because of the emerging economic consequences of the pandemic, the federal government set up a task force which, among other things, took on the regular monitoring of the situation on the apprenticeship market. As one of these measures, the cantons reported monthly on the apprenticeship contracts signed and compared these with the number of contracts signed in the same month of the previous year. In this way, the situation on the apprenticeship market could be monitored at least monthly, starting from April 2020 onwards.

**Fig. 1:**
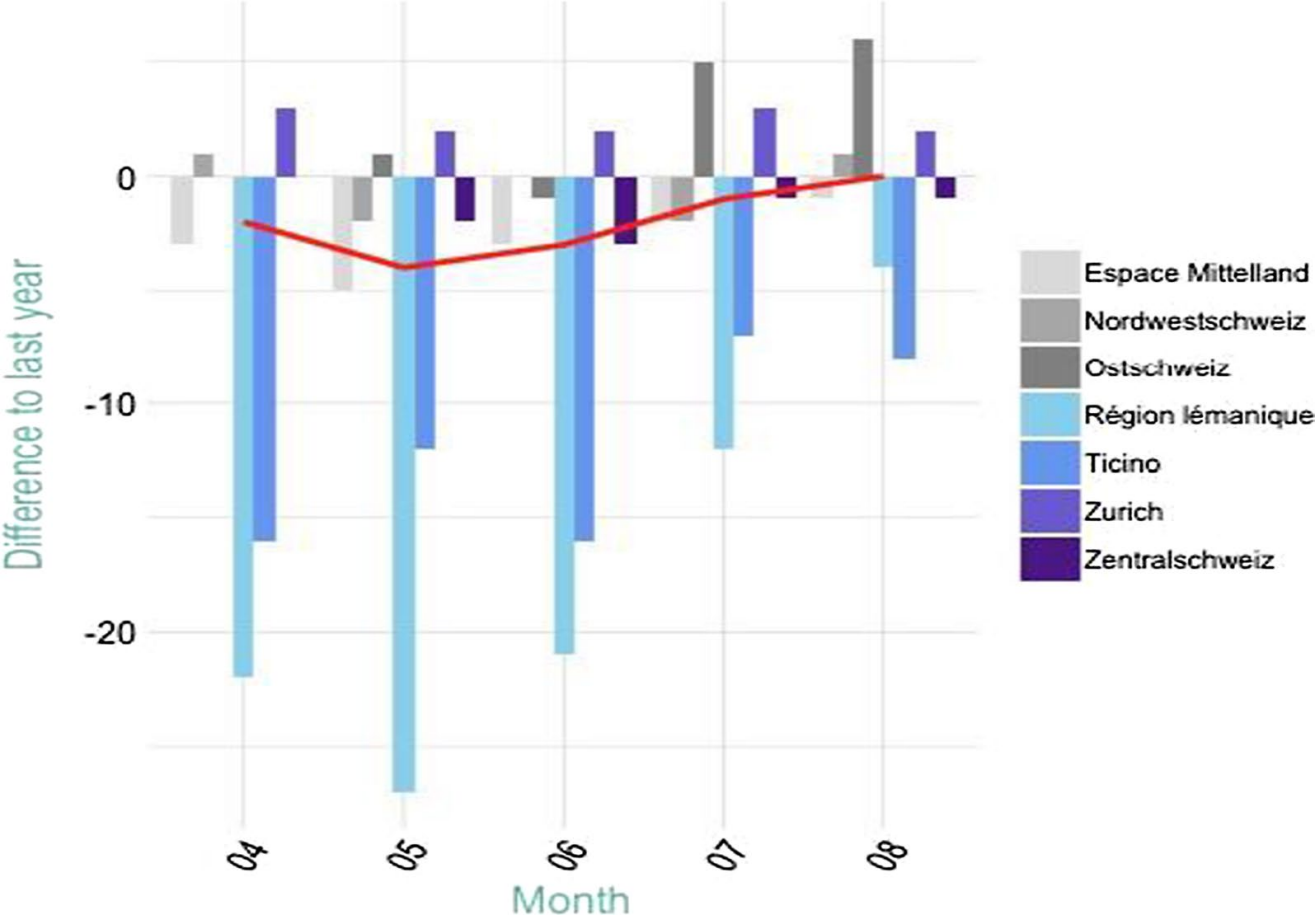
Difference in signed apprenticeship contracts compared to previous year. Differences in signed contracts compared to previous year by NUTS-2 regions. The red solid line represents the Swiss average. *Data source*: “Taskforce Perspektive Berufslehre 2020”

Looking at these figures (Fig. 1), one can see that the number of signed apprenticeship contracts declined in the first half of the year 2020 relative to 2019, but also a catch-up effect reaching the same number of apprenticeship contracts by August 2020 compared to the previous year. The substantial differences in signed contracts between “Nomenclature of Territorial Units for Statistics-2” (NUTS-2) regions can mainly be related to differences in the timing of the conclusion of the contracts. Traditionally, contracts are already signed as early as October in the year prior to the start of the apprenticeship in the German speaking cantons, whereas in the French and Italian speaking part of the country firms and students wait till just a few months before the start of the apprenticeship.^3^ Thus, while for the German-speaking cantons a major part of contracts was already signed before the COVID-19 outbreak, this process just started in *Région lémanique* and *Ticino*, which Fig. 5 in “Appendix” documents for April 2020.

### Demand for apprentices

Regarding the demand for apprentices alone, there are concurrent indications that the number of offered positions to start the apprenticeship in 2020 did not change substantially compared to previous years. First, as Fig. 1 documents, the number of signed apprenticeship contracts reached the previous years’ numbers by the end of August 2020. Thus, it is fair to assume that the total demand for apprentices did not change. Second, the “Nahtstellenbarometer” of the SERI monitors the training companies twice a year in April and in August. These representative surveys showed that the firms’ demand for apprentices had not changed in 2020 relative to 2019. In August 2020, most of the companies reported, that they had not changed their number of apprenticeship positions in 2020, and the share of companies that have offered more or fewer had hardly changed. More specifically, the number of firms that had reduced their numbers of positions was unchanged compared to the previous years (2020: 8%; 2019: 8%; 2018: 9%).

Third, the SERIs “Taskforce Perspektive Berufslehre” reported monthly from April 2020 onwards the signed contracts and open apprenticeship positions registered. In Table 1 we contrast the number of open positions and signed contracts in the years 2020 to 2021. It is noteworthy that the number of vacancies in 2020 is very similar to the number in 2021 and the monthly changes in the numbers of vacancies and the number of signed contracts show the same ratios in both years. While there is not every apprenticeship offer registered we can be sure that there was no lack of open positions, which could justify the difference in signed contracts in Fig. 1. Fourth, the private provider Yousty issued a press release in April 2020 stating that the number of open positions barely changed since the beginning of the COVID-19 outbreak.^4^

**Table 1:** Open apprenticeships (total) and signed contracts (cumulative), 2020 and 2021. *Source*: “Taskforce Perspektive Berufslehre 2020”. Numbers are recorded at the end of the respective month. The monthly changes in the number of vacancies and signed contracts are not equal, because the number of vacancies does not cover all vacancies, but only those posted on the national platform

	2020		2021	
	Open positions	Signed contracts	Open positions	Signed contracts
April	22,195	42,212	21,083	43,106
May	19,271	47,811	17,889	49,642
June	16,221	55,454	14,502	57,946

Therefore, as there are no signs that the demand for apprentices changed much in the aftermath of the shutdown in March 2020, the focus of this work is to investigate whether the supply side in the Swiss apprenticeship market could explain this striking pattern of an initial decline in signed apprenticeship contracts and subsequent catch-up.

### Supply of apprentices

The supply side of prospective apprentices consists mainly of graduates of compulsory school, but also those entering the apprenticeship market after an interim solution, youth re-orienting after having dropped out of general education or even those intending to train for a second occupation after having already successfully completed an apprenticeship or a general education. For them, applying to occupations became more difficult during the COVID-19 pandemic.

From the perspective of these individuals, the situation they were confronted with was unique in several aspects. First, with the classification of the evolving COVID-19 pandemic as “exceptional situation”, the daily life of youth changed radically. It required significant sacrifice and acclimatisation, e.g., to a life of closed schools and distance learning. Second, there was great uncertainty about the length and intensity of this crisis. Applying for vacant apprenticeship posts may not be advisable, as the firms might run into financial problems or might not be able to provide a full vocational education and training for other reasons. Third, as many businesses were forced to close their operations or work from home during the shutdown, the application process became difficult and complicated, so much so that many potential applicants tended not to apply at all. Fourth, although this was more of concern for the cohort looking for an apprenticeship in 2021, the usual training fairs and information days in the companies, as well as trial (apprenticeships) working days could not take place. Young people were therefore not able to inform themselves about potential employers and job profiles as was the practice in previous years. Putting all this together, it is quite realistic to assume that the supply of learners has been affected by the COVID-19 pandemic.

## Data and methodology

**Fig. 2:**
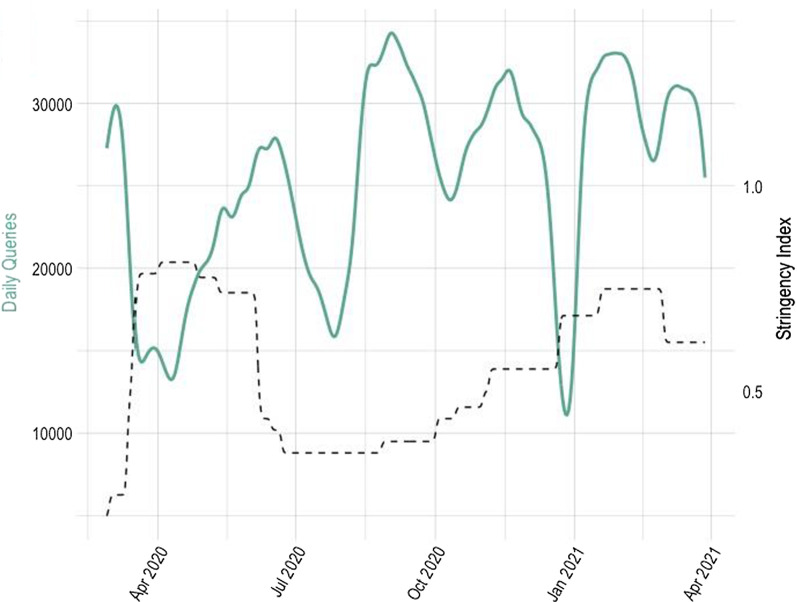
Total daily queries and Stringency Index. Green solid line represents the daily queries, Nadaraya-Watson Kernel-smoothed (left axis), the black broken line is the Stringency Index (right axis), exemplarily for the canton Zurich

Our data base consists of every user query searching for an apprenticeship on the public national online apprenticeship platform from the 28th February 2020 until the 04th April 2021.^5^ This official platform operates on behalf of the Swiss cantons and is not only the main source for apprenticeship vacancies in Switzerland, but also the official Swiss information portal for study and career guidance. People interested in starting an apprenticeship can search in all four national languages by occupation and location for open apprenticeships. While not every apprenticeship position is advertised online and there are other (private) online platforms, this is the most comprehensive and a well-known outlet for apprenticeship advertisements, guaranteeing representativeness of the data. In the investigated 13 months we observe over 10 million search queries.^6^ Figure 2 shows how the number of daily search queries evolved over this time. The major advantage of our measure of apprentices supply, even though we do not know the number of potential apprentices, is that we observe revealed preferences on a daily basis, with those interested in apprenticeships sending search queries. Further, they stop searching as soon as they found an apprenticeship or lost interest (also temporarily) for other reasons, which gives us a good proxy for the daily interest in apprenticeship training positions, by field of occupation and region. Other measures are mainly only available on a yearly basis or as rough proxies.

The (weekly) average of daily queries one and two weeks before and after the first shutdown on March 16th, 2020, are shown in Table 2. We observe decreasing numbers of daily search queries in 2020 accompanied by an increasing Stringency Index, a measure of toughness of the politically imposed restrictions in response to the pandemic—which is described in more detail in the following. For 2021 both, daily queries and the Stringency Index are rather on a constant level comparable to the “pre-crisis” level.

**Table 2:** Daily queries before and after the first shutdown, 2020 vs. 2021

	2020		2021	
	Daily queries (7 days average)	Stringency Index	Daily queries (7 days average)	Stringency Index
March, 02.–08.	30,801	0.250	31,005	0.620
March, 09.–15.	26,971	0.349	30,936	0.620
March, 17.–23.	13,261	0.771	30,771	0.620
March, 24.–30.	15,164	0.787	29,035	0.620

Averages of daily queries in week 2 and week 1 before and after the date of the first shutdown, March 16th, 2020. The Stringency Index is a measure for restrictiveness of politically imposed countermeasures, which is described later in this section

For the further analysis, we aggregate the individual queries by day, canton, and occupation category, leading to a total of 512,148 observations.^7^ To measure the effect of the shutdown policy on the number of search queries we focus on two different types of variables of interest. First, a binary variable is constructed which is equal to 1 starting with the national shutdown from the 16th March, 2020 onwards until the (second) easing of the national restrictions on the 11th May, 2020.^8^ To investigate the second (national) shutdown a binary variable is created which equals 1 for the days from the 22nd December 2020 until 28th February 2021. Second, we use the “*KOF Stringency Index*/*KOF Stringency Index Plus*” (Pleninger et al., 2021), which are constructed as composite measures of the different restrictions and are ranging from 0 (no restrictions) to 1 (full shutdown).^9^

The availability of the KOF Stringency Index on a cantonal base gives us the advantage that we can capture not only the impact of variations of this index over time but also between geographical regions, as in Switzerland, like in other countries, different levels of restrictions were in function for different regions at the same time. The construction of the daily KOF Stringency Indices follows closely the *Oxford Covid-19 Government Response Tracker*, but refines the measure to account for cantonal differences within Switzerland. Figure 2 shows the Stringency Index over the investigated time period exemplary for the canton of Zurich.

To investigate if the effect of the restrictions is (linearly) declining or growing over time, a variable “Stringency over time” is constructed as follows:

$$Stringency over time=Stringency \times \frac{t}{100},$$

where t = 1,…,T is an increasing numbered index for the respective day, i.e. is equal to 1 for the 28th February 2020, up to 402 for the 4th April 2021. Dividing by 100 leads to more interpretable coefficients, which can now be seen as for every 100 days the Stringency Index coefficient is changed by the Stringency over time coefficient.^10^

To control for specific (COVID-19-unrelated) patterns, we construct several variables. Repeated weekly patterns are accounted for by including binary variables for each day of the week, while seasonal variations are captured using monthly indicators. For general cantonal differences canton dummies are included, as well as dummies to account for the occupation group in which the search was done. Since search intensity is generally lower on public holidays, as well as school vacations those are also included on a cantonal level. To take into account that apprenticeship contracts are concluded at different times in the year, depending on the region we include the already signed apprenticeship contracts as share of the previous years total number as an additional control variable. Descriptive statistics on some of these variables can be found in Table 3.

**Table 3:** Descriptive statistics

	Mean	SD	Min	Max
*Outcomes*				
# Queries by day	25,386.61	11,513.79	3259	52,105
# Queries by day, canton and occupation	14.520	56.787	0	7353
Log(# Queries by day, canton and occupation)^a^	1.381	1.442	0	8.903
*Covariates*				
Stringency Index	0.559	0.179	0.194	0.815
Stringency plus Index	0.554	0.167	0.175	0.750
Stringency over time^b^	1.153	0.842	0.002	2.753
Public holidays	0.025	0.157	0	1
School vacation	0.256	0.437	0	1
Share signed apprenticeship contracts^c^	0.709	0.271	0.001	1

512,148 Observations. *SD* standard deviation

^a^To avoid NA values due to undefined log(0) we chose to add + 1 to each value before log-transformation

^b^Stringency Index multiplied by an in time increasing variable as described in the text

^c^Due to missing information in the monitoring 389,844 observations

In the following we analyse the relation between the shutdown and restriction variables and the number of search queries in a linear regression framework. To check the dependence of the results on the choice of the method in “Appendix”, the main results are replicated with the Stringency plus Index, as well as using Pseudo-Poisson Maximum Likelihood (Santos Silva & Tenreyro, 2006).

## Results

To investigate the influence of the restrictions imposed due to the COVID-19 pandemic on the supply of apprentices, we analyse the correlation of the Stringency Index and the shutdowns on the number of daily search queries by canton and occupation. Important for the interpretation of the coefficients is that the outcome, the number of daily search queries, is used in a log-transformation. Thus, for an increase of 1 unit in the Stringency Index we observe 61.7% less search queries in Panel A of Table 4, column (1). Though, it is important to keep in mind that an increase from 0 to 1 in the stringency would translate to a switch from no restrictions to the maximum possible restrictions (the maximum observed Stringency Index value in the data was 0.815). To put this index into perspective, we have seen that the first shutdown in March 2020 resulted in an increase of about 0.5 units.^11^

**Table 4:** Main results

	(1)	(2)	(3)	(4)
*Panel A: Stringency Index*				
Stringency Index	− 0.617*** (0.014)	− 0.683*** (0.015)	− 0.579*** (0.017)	− 0.569*** (0.017)
Stringency over time		0.091*** (0.012)		
Public holiday	− 0.304*** (0.009)	− 0.303*** (0.009)	− 0.277*** (0.009)	− 0.280*** (0.009)
School vacation	− 0.208*** (0.006)	− 0.209*** (0.006)	− 0.200*** (0.006)	− 0.187*** (0.006)
Share signed apprentice. contr				− 0.604*** (0.059)
Observations	512,148	512,148	236,964	236,964
Time range	28.02.’20–04.04.’21		28.02.’20–31.08.’20	
*Panel B: Shutdowns*				
Stringency Index		− 0.095*** (0.019)	− 0.191*** (0.022)	
Stringency over time			0.053** (0.020)	
Shutdown 1	− 0.394*** (0.006)	− 0.366*** (0.007)	− 0.378*** (0.008)	− 0.379*** (0.006)
Shutdown 2				− 0.199*** (0.007)
Public holiday	− 0.326*** (0.009)	− 0.324*** (0.009)	− 0.328*** (0.009)	− 0.277*** (0.008)
School vacation	− 0.206*** (0.006)	− 0.207*** (0.006)	− 0.208*** (0.006)	− 0.171*** (0.006)
Observations	512,148	512,148	512,148	512,148
Time range	28.02.’20–04.04.’21			

Outcome is the log (daily queries per canton and occupation). Other controls are dummies for: Days of the week, month, occupation, canton, year. Shutdown 1 is defined as 16.03–11.05.2020. Shutdown 2 is defined as 22.12.2020–28.02.2021. Columns (3) and (4) in Panel A are restricted until apprenticeships 2020 started in the beginning of September. Standard errors in parentheses are clustered standard errors on occupation x canton level. **, and *** marks statistical significance at the 1%, and 0.1% level

Estimates for the whole sample in Panel A, column (1), as well as column (3) for the time range until the apprenticeships 2020 started in the beginning of September, are robust, with barely changing coefficients of − 0.617 and − 0.579, respectively. Putting these numbers into perspective, for the first shutdown in March 2020 this translates to a decrease of about 30% of search queries. This is in the range of the decrease in search queries during public holidays (with coefficients between − 0.277 and − 0.328), and larger in magnitude compared to the effect of school vacations (with coefficients between − 0.171 and − 0.209).

To investigate the effect of the restrictions over time (column (2) in Table 4, Panel A) includes the *Stringency over time* variable, showing that for each additional 100 days, the correlation between the Stringency Index and the number of search queries becomes smaller in effect by 0.091. Therefore, after 1 year the effect of the Stringency Index measured restrictions on the daily search queries is halved.

Although, the share of signed apprenticeship contracts in the respective NUTS-2 region has the expected negative impact on search activities (Panel A of Table 4, column (4)), there is almost no change in the effect of the restriction index on daily search queries. In other words, the restrictions themselves affected the search activities of those still in search of an apprenticeship at the time the restrictions were imposed, in a similar magnitude irrespective of the number of outstanding contracts at that time in their region.

Panel B in Table 4 reports the conditional correlation for the first shutdown (*Shutdown 1*) in column (1), which can be interpreted as 39.4% less search queries during the first shutdown. This is a larger decrease as suggested by the Stringency Index discussed before, not only capturing the first shutdown, but changing throughout the full observation period. Controlling for the first shutdown, in Panel B, column (2) leads to a substantial decrease of the correlation between the Stringency Index and the daily search queries compared to Panel A, column (1). Among the containment policies during the full time of the first shutdown was the uniquely used closure of obligatory schools. If this reduction in the influence of the equally weighted stringency measure on search queries was due to the closures of obligatory schools, if the negative relation between containment policies and search activity diminishes over time for other reasons, or if both played a role cannot be finally answered.

Even though on a lower magnitude, column (3) in Table 4s’ Panel B we still find the decreasing correlation, with a positive *Stringency over time* coefficient (0.053). For the second shutdown (*Shutdown 2*, Table 4, Panel B in column (4)) we observe a decrease in search queries of about 20%. This lower effect is also visible if we investigate the shutdowns descriptively on a raw, daily basis in Figs. 7 and 8 in “Appendix 1”. We find a clear effect for *Shutdown 1*, regenerating slowly, while for *Shutdown 2* we observe a short and strong effect, which is partly explainable by school vacations and the public holidays around Christmas and New Year. Defining different lengths of the Shutdown 1, Fig. 6 in “Appendix” shows that the effect was strongest two weeks after the shutdown on the 16th of March and steadily decreased afterwards.

Tables 5 and 6 in “Appendix 2” investigate the sensitivity of those results regarding the *Stringency* vs. *Stringency plus* Index, and the estimation method using the PPML method. The results are comparable to our main results in Table 4 and the conclusions drawn do not change.

**Fig. 3:**
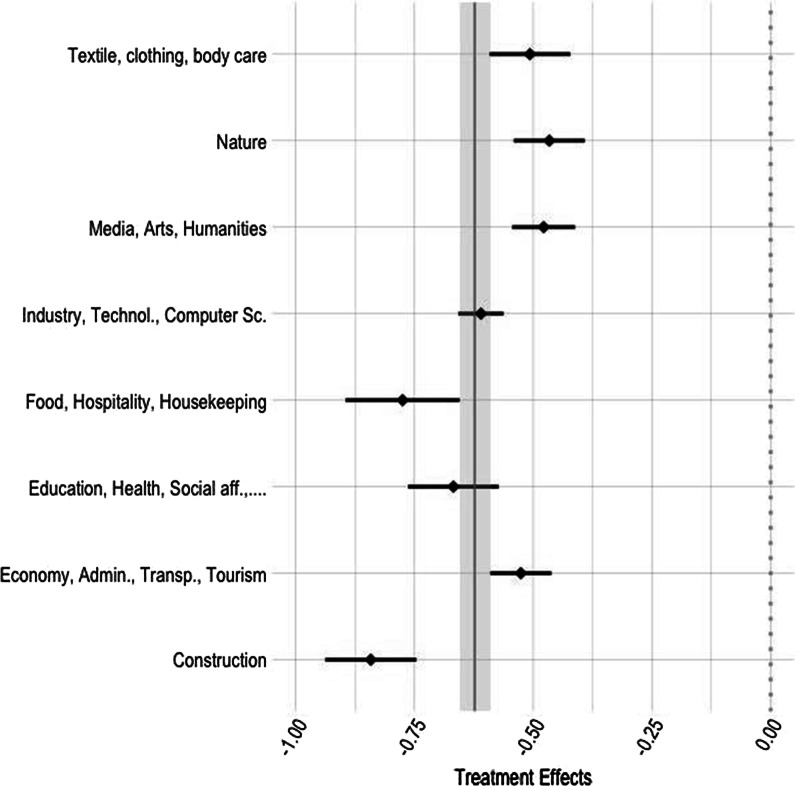
Treatment effect by occupation, 8 categories. Correlation of Stringency Index and daily queries; linear model—specification as in Table 4, Panel A, column (1). Blue diamonds are the point estimates for the specific region accompanied by the 95% confidence interval. Vertical solid grey line (with grey shaded confidence interval) is the population average effect. Eight categories according to the SwissDoc classification

**Fig. 4:**
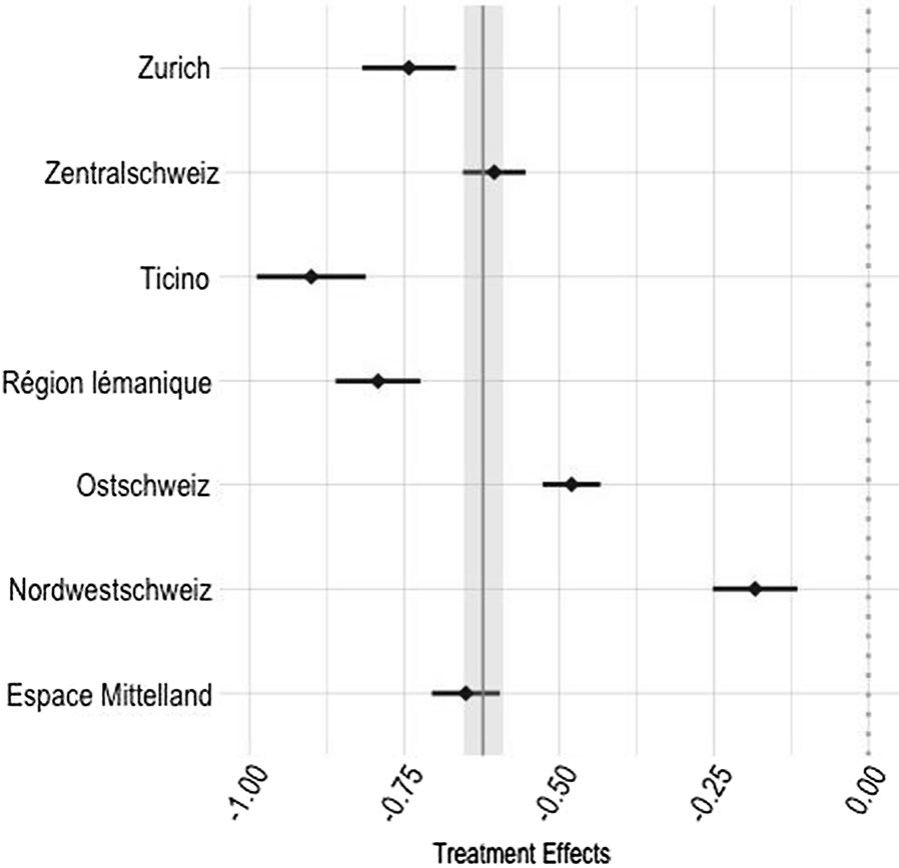
Treatment effects by NUTS-2 regions. Correlation of Stringency Index and daily queries; linear model—specification as in Table 4, Panel A, column (1). Blue diamonds are the point estimates for the specific region accompanied by the 95% confidence interval. Vertical solid grey line (with grey shaded confidence interval) is the population average effect

**Fig. 5:**
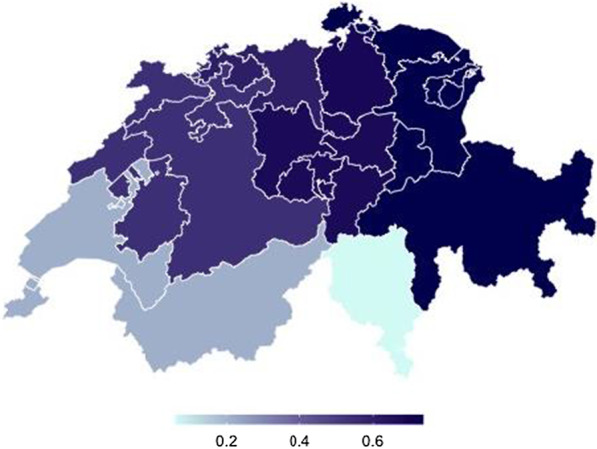
Share of signed apprenticeship contracts, April 2020. In April 2020 as a share of the number of previous years’ final number of signed apprenticeship contracts within a NUTS-2 region

**Fig. 6:**
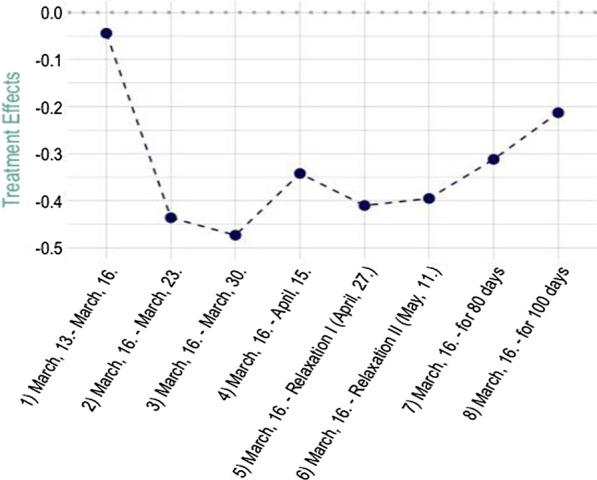
Shutdown 1 effects for different time periods. Treatment effects from separate regressions using different definitions, i.e., time spans of the treatment variable. Since (canton by occupation clustered) standard errors are very small (< 0.006) they are omitted in this illustration

**Fig. 7:**
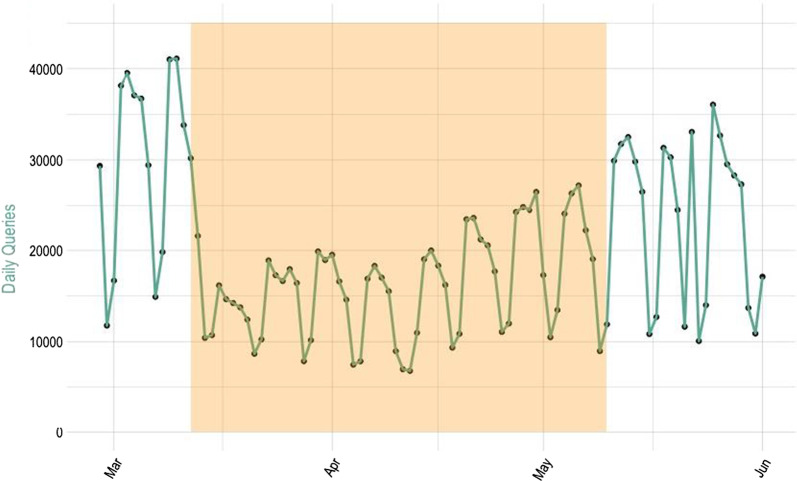
First shutdown, daily queries, Feb 28–Jun 01, 2020. Green solid line connects for illustration purposes the daily queries (black points). Yellow shaded area represents the time between 13.03 and 11.05.2020

**Fig. 8:**
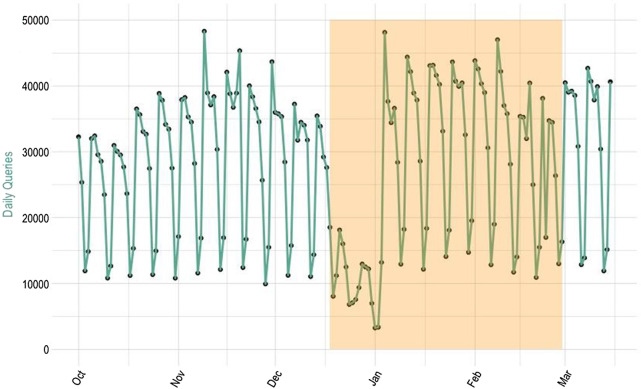
Second shutdown, daily queries, Oct 01, 2020–Mar 15, 2021. Green solid line connects for illustration purposes the daily queries (black points). Yellow shaded area represents the time between 22.12.2020 and 28.02.2021

To investigate the robustness of the presented estimates, we provide subgroup estimates by types of occupations and NUTS-2 regions. Figure 3 investigates groups defined by eight occupation categories and shows different occupation types to react very similar to the restrictions.^12^ Although effects differ by occupation categories, they are all statistically significant, negative, and similar in magnitude. Figure 4 sheds some light on differential effects by regions. The strongest effect is visible for the regions of *Ticino* and *Région lémanique*, which are also the regions first and, at least in the beginning of the pandemic, hardest affected by the Coronavirus. As discussed before, in these regions, apprenticeship contracts are signed closer to the start of the apprenticeship. Results in Fig. 4 confirm this as the search intensity in these regions is reduced most. Despite the results are heterogeneous, it can be noted that all the regions show significant and negative responses to the stringency of restrictions.

## Conclusion and discussion

This paper examines the impact of the economic, social, and educational restrictions triggered by the COVID-19 pandemic on the behaviour of apprenticeship supply before, during and after these shutdowns. The behaviour of supply is analysed using a novel dataset formed by around 10 million searches on the official public platform for apprenticeships. With this data, the search intensity for apprenticeships can be approximated and tracked daily. The analysis shows a strong temporary decline in search queries and therefore assumingly also of the supply of apprentices, during the first shutdown in mid-March 2020. The further development of the search queries correlates strongly with the KOF Stringency Index, which shows the degree of restrictions imposed daily and per canton. Furthermore, the analyses also show that the influence of the restrictions on search intensity decreases substantially over time. Although we are far from claiming causality for the relationship between the search queries and the number of signed apprenticeship contracts per month, it is striking that first the slowing and then the accelerating of the number of signed contracts between April and October 2020 coincides with the temporal pattern of search intensity just before these months on the national platform for apprenticeship vacancies. With regard to the development of the Swiss apprenticeship market during the 2020 pandemic year, the analyses provide therefore an explanation for the initial decline in apprenticeship contract signings at the beginning of the first shutdown and the subsequent catch-up effect.

However, due to several limitations in the availability of data and the uniqueness of certain important pandemic-related restrictions, the findings are limited in at least three respects. The answer to these three questions is therefore the subject of future research.

Firstly, we do not have similarly disaggregated data on the demand for apprentices available at high temporal frequency for the period considered here. Even if we have individual data points for this period that are sufficient as an indication to assume that the demand for apprentices had not changed negatively after the first shutdown and also after the second shutdown, we cannot observe supply and demand simultaneously.

Secondly, we cannot separately examine the impact of school closures on search intensity because compulsory schools were only closed once during the first shutdown and in all cantons simultaneously. Even though it is very likely that the school closures had a particularly dampening influence on search intensity, and this would therefore also explain why later fluctuations in the Stringency Index no longer had such a large influence on search intensity, other explanations cannot be ruled out. On the one hand, and as with other behaviours such as geographic mobility, it may be that the same restriction measures have no longer elicited the same responses from the population over time, a phenomenon known as COVID-19-fatigue. In our case, this could also be interpreted as meaning that the young people had recovered from an initial "shock" and knew afterwards how to deal with the crisis. On the other hand, state support measures could also have had an effect, by means of which school leavers in particular were motivated to actively seek apprenticeships again and in some cases also supported them in doing so. This would also explain the increase in search activities that was already evident towards the end of the first shutdown.

Thirdly, and beyond the scope of our analysis, the question remains as to why the decline in GDP of around three per cent in 2020, in contrast to much smaller crises in the past, did not leave a trace on the apprenticeship market. We can only speculate about this at present. The only certainty is that rarely in economic history has a recession started on a very specific day and ended after a very specific period of time, as decreed by the authorities, and at the same time accompanied by fiscal support measures that know no comparison in history. However, it can be deduced from these correlations that the timing of the exogenous shock (COVID-19 pandemic), namely at a time when more than 60% of apprenticeship contracts for 2020 had already been signed, as well as the timing of the transition to reopened schools contributed to the fact that the economic slump in 2020 bypassed the apprenticeship market without leaving noticeable traces.

Finally, for the future monitoring of and research on the apprenticeship market, it can be concluded from the analyses that the data on the search activities for apprenticeship positions provide a very good high-frequency indicator to follow the development on the supply side of the apprenticeship market in "real time".

## Data Availability

The data that supports the findings of this study are available from the SDBB|CFSO but restrictions apply to the availability of these data, which were used under license for the current study, and so are not publicly available. The KOF Stringency Index is available from the KOF High Frequency Dashboard (https://kofdata.netlify.app/#/).

## Appendix 1: Additional resources

See Figs. 5, 6, 7 and 8.

## Appendix 2: Additional results

See Tables 5 and 6.

**Table 5:** Main results; Stringency plus

	(1)	(2)	(3)	(4)
*Panel A: Stringency Index*				
Stringency plus	− 0.664*** (0.015)	− 0.733*** (0.016)	− 0.620*** (0.019)	− 0.610*** (0.018)
Stringency plus over time		0.094*** (0.012)		
Public holiday	− 0.304*** (0.009)	− 0.303*** (0.009)	− 0.279*** (0.009)	− 0.281*** (0.009)
School vacation	− 0.210*** (0.006)	− 0.210*** (0.006)	− 0.200*** (0.006)	− 0.187*** (0.006)
Share signed apprentice. contr				− 0.605*** (0.059)
Observations	512,148	512,148	236,964	236,964
Time range	28.02.’20–04.04.’21		28.02.’20–31.08.’20	
*Panel B: Shutdowns*				
Stringency plus		− 0.086*** (0.020)	− 0.173*** (0.024)	
Stringency plus over time			0.063** (0.021)	
Shutdown 1	− 0.394*** (0.006)	− 0.371*** (0.007)	− 0.386*** (0.008)	− 0.379*** (0.006)
Shutdown 2				− 0.199*** (0.007)
Public holiday	− 0.326*** (0.009)	− 0.324*** (0.009)	− 0.329*** (0.009)	− 0.277*** (0.008)
School vacation	− 0.206*** (0.006)	− 0.207*** (0.006)	− 0.208*** (0.006)	− 0.171*** (0.006)
Observations	512,148	512,148	512,148	512,148
Time range	28.02.’20–04.04.’21			

Outcome is the log (daily queries per canton and occupation). Other controls are dummies for: Days of the week, month, occupation, canton, year. The Stringency over time coefficient is estimated on a basis of 100 days. Shutdown 1 is defined as 16.03.–11.05. 2020. Shutdown 2 is defined as 22.12.2020–28.02.2021. Columns (3) and (4) in Panel A are restricted until apprenticeships 2020 started in the beginning of September. Standard errors in parentheses are clustered standard errors on occupation x canton level. ***Marks statistical significance at the 0.1% level

**Table 6:** Main results, PPML

	(1)	(2)	(3)	(4)
*Panel A: Stringency Index*				
Stringency Index	− 0.959*** (0.022)	− 0.917*** (0.003)	− 1.032*** (0.029)	− 1.031*** (0.029)
Stringency over time		0.058** (0.021)		
Public holiday	− 0.504*** (0.113)	− 0.506*** (0.113)	− 0.317* (0.160)	− 0.317* (0.161)
School vacation	− 0.383*** (0.009)	− 0.382*** (0.009)	− 0.380*** (0.016)	− 0.376*** (0.011)
Share signed apprentice. contr				− 0.076 (0.205)
Observations	512,148	512,148	236,964	236,964
Time range	28.02.’20–04.04.’21		28.02.’20–31.08.’20	
*Panel B: Shutdowns*				
Stringency Index		− 0.485*** (0.028)	− 0.467*** (0.032)	
Stringency over time			0.054*** (0.006)	
Shutdown 1	− 0.497*** (0.010)	− 0.340*** (0.013)	− 0.367*** (0.014)	− 0.471*** (0.010)
Shutdown 2				− 0.369*** (0.020)
Public holiday	− 0.546*** (0.113)	− 0.530*** (0.113)	− 0.529*** (0.113)	− 0.461*** (0.118)
School vacation	− 0.385*** (0.009)	− 0.385*** (0.009)	− 0.386*** (0.009)	− 0.325*** (0.010)
Observations	512,148	512,148	512,148	512,148
Time range	28.02.’20–04.04.’21			

Outcome is the daily queries per canton and occupation. Other controls are dummies for: Days of the week, month, occupation, canton, year. The Stringency over time coefficient is estimated on a basis of 100 days. Shutdown 1 is defined as 16.03.–11.05.2020. Shutdown 2 is defined as 22.12.2020–28.02.2021. Columns (3) and (4) in Panel A are restricted until apprenticeships 2020 started in the beginning of September. *, **, and ***Marks statistical significance at the 5%, 1%, and 0.1% level

## Abbreviations

VET: Vocational education and training
SDBB|CFSO: Swiss service centre for vocational education and training
SERI: Swiss State Secretariat for Education, Research, and Innovation
COVID-19: New coronavirus
NUTS-2: Nomenclature of Territorial Units for Statistics

## Footnotes

1 Contrary to hiring regular workers, apprenticeships are long-term investments of (training) companies as they come with contracts that last either three or four years. Therefore, for most companies the decision to offer training positions is a strategic one, followed by a time-consuming process of advertising, interviewing, and selecting the right candidates. Bearing this in mind, it is rather unlikely that interested companies would have withdrawn their advertised vacancies only to put them again on the market some weeks later or replaced them by new ones in such a short time.

2 The Swiss Journal of Economics and Statistics, among other journals, started a special focus with many more articles on the economic outcomes of the COVID-19 pandemic in 2020.

3 The latter is a consequence of the fact that most students prefer a general education and therefore wait to see whether they will gain access to this education or not. Only if they do not make it do they look for an apprenticeship.

4 Sources are «Nahtstellenbarometer»: https://cockpit.gfsbern.ch/de/cockpit/nahtstellenbarometer-2020-3/; «Perspektive Berufslehre»: https://taskforce2020.ch/de/monitoring; Yousty April 2020 press release: https://www.yousty.ch/de-CH/Pressemitteilungen. (In German; accessed 23.07.2021).

5 berufsberatung.ch (de; fr: orientation.ch; it: orientamento.ch) is the official platform for information about and searching for apprenticeships offered by the SDBB | CFSO, which is an agency of the Swiss Conference of Cantonal Ministers of Education. Although there are also private platforms and there are other ways to search for apprenticeships, the national platform that collects data on vacancies in all cantons is probably the platform that most apprenticeship seekers will consult during their search and application process at least once.

6 From the initial 12 million search queries, we removed all search queries in Liechtenstein. We removed those not specifying an occupation or location, which is needed to investigate containment policies on a cantonal level. Further, we removed all automated (crawler/bot) queries, leading to a final set of about 10 million search queries. To set this number into perspective: the 10 million queries result not only from individuals interested in an apprenticeship position. Parents, teachers, firms, administrations, and career guidance centres gather information there for advising the potential apprentices. Still, the search for a suitable apprenticeship is complex and requires searching several times on several days until a contract is signed or interest in training is lost. During the COVID-19 crisis (no trial apprenticeships, no information days, etc.) individuals searching for an apprenticeship were more dependent than usual on the help of confidants and institutions. Thus, even though we cannot distinguish the senders of search queries, if having a bias, we expect the estimated effect to be a conservative, lower bound effect.

7 We chose to use the subgroups of the SwissDoc-number classification, resulting in 49 occupational groups. 402 (days) × 26 (cantons) × 49 (occupations) = 512,148 observations.

8 To investigate if the effect is driven by the definition of this variable, as well as to see how the effect evolved over time, we chose to add various other definitions, i.e., for 7, 14, 30, 80 and 100 days, as well as until the first easing of the restriction (25th April, 2020).

9 It combines 9 indicators to an equally weighted one-dimensional index: school closures, workspace closures, cancellation of public events, closure of public transport, restrictions on gatherings, requirements to stay at home, internal movement restrictions, international travel controls and public info campaigns. The “plus” variant of the Stringency Index adds a tenth, i.e., facial coverings, as well as refines workplace closures to incorporate reduced opening hours. For the detailed construction of the indices, we refer to Pleninger et al. (2021) and the documentations provided there.

10 The analysis of the time-dependency of the Stringency Index on outcomes is motivated by the observation of a COVID-19 shutdown fatigue. Goldstein et al. (2021) e.g., found that there is a fading effect of the COVID-19 related restrictions on mobility and health outcomes, using a panel of about 150 countries.

11 An increase in 0.5 is due to a bundle of imposed or tightened restrictions. Some more concrete examples are: Closing all schools and universities increases the *Stringency Index* by 0.111, while allowing gatherings of 50 instead of 10 people decreases the index by 0.028.

12 The eight occupation categories are chosen according to the super-categories in the official SwissDoc classification.
